# Predictors of Colorectal Polyp Recurrence after the First Polypectomy in Private Practice Settings: A Cohort Study

**DOI:** 10.1371/journal.pone.0050990

**Published:** 2012-12-03

**Authors:** Jean-François Viel, Jean-Marie Studer, Yves Ottignon, Jean-Pierre Hirsch, Franche-Comté Polyp Surveillance Study Group

**Affiliations:** 1 Department of Epidemiology and Public Health, University Hospital, Rennes, France; 2 Private gastroenterology practice, Montbéliard, France; 3 Private gastroenterology practice, Besançon, France; 4 Private gastroenterology practice, Besançon, France; The University of Texas M. D. Anderson Cancer Center, United States of America

## Abstract

**Background:**

Supplementary observational data in the community setting are required to better assess the predictors of colorectal polyp recurrence and the effectiveness of colonoscopy surveillance under real circumstances.

**Aim:**

The goal of this study was to identify patient characteristics and polyp features at baseline colonoscopy that are associated with the recurrence of colorectal polyps (including hyperplastic polyps) among patients consulting private practice physicians.

**Patients and Methods:**

This cohort study was conducted from March 2004 to December 2010 in 26 private gastroenterology practices (France). It included 1023 patients with a first-time diagnosis of histologically confirmed polyp removed during a diagnostic or screening colonoscopy. At enrollment, interviews were conducted to obtain data on socio-demographic variables and risk factors. Pathology reports were reviewed to abstract data on polyp features at baseline colonoscopy. Colorectal polyps diagnosed at the surveillance colonoscopy were considered as end points. The time to event was analyzed with an accelerated failure time model assuming a Weibull distribution.

**Results:**

Among the 1023 patients with colorectal polyp at baseline, 553 underwent a surveillance colonoscopy. The mean time interval from baseline colonoscopy to first surveillance examination was 3.42 (standard deviation, 1.45) years. The recurrence rates were 50.5% and 32.9% for all polyps and adenomas, respectively. In multivariate models, the number of polyps at baseline was the only significant predictor for both polyp recurrence (hazard ratio [HR] 1.19, 95% CI 1.06 to 1.33), and adenoma recurrence (HR 1.17, 95% CI 1.03 to 1.34).

**Conclusion:**

The efficacy of surveillance colonoscopy in community gastroenterology practice compared favorably with academic settings. This study provides further evidence that the number of initial colorectal polyps is useful for predicting the risk of polyp recurrence, even in the community setting.

## Introduction

Most colorectal cancers (CRCs) are believed to develop through a complex multistep process from normal mucosa to benign adenoma and then to carcinoma. Interrupting this adenoma-carcinoma sequence with coloscopy and polypectomy is thus recommended to prevent CRC. Periodic surveillance examinations are considered necessary after the removal of colorectal polyps because of the risk of newly discovered lesions. Patients with a clean colon after initial polypectomy have a higher risk of developing adenomas than non-polyp carriers [1]. Missed and incompletely excised lesions are thought to be likely explanations for some of the adenomas found during surveillance, even though the examinations are performed by experts with extensive colonoscopic experience [2]. Adenoma recurrence rates vary greatly between studies (approximately 20%–50% within 3–5 years, see reviews [3]–[5]), due to differences in patient characteristics at baseline, the follow up duration, patient compliance, inclusion criteria, and the quality of the initial colonoscopy and polypectomy.

Many studies have suggested that some characteristics present at index colonoscopy confer a high risk of advanced adenoma recurrence at surveillance colonoscopy: 3 or more adenomas (pooled relative risk [RR] 2.52, 95% confidence interval [CI] 1.07 to 5.97), and adenomas with high-grade dysplasia (pooled RR 1.84, 95% CI 1.06 to 3.19) [6]. Professional groups have consequently developed guidelines for surveillance colonoscopy intervals after polypectomy, introducing the concept of “risk stratification” with the goal of more efficient detection of advanced adenomas or early cancers.

Most of the surveillance studies included selected (male, more educated, and more affluent) patients and were conducted in academic settings. Under real circumstances in clinical practice, the effectiveness of colonoscopy could be substantially less clear than in the academic setting [7], and even lower in a private practice model under which physicians face additional factors, including financial and time pressure. Supplementary observational data in the community setting regarding the findings from surveillance colonoscopies and the subsequent patient outcome are therefore required to better evaluate the effectiveness of surveillance examinations [3].

In a decisively pragmatic approach, the goal of this study was to identify patient characteristics and polyp features at baseline colonoscopy that are associated with the recurrence of colorectal polyps (including hyperplastic polyps) among patients consulting private practice physicians.

## Methods

### Setting and Study Population

Participants were prospectively enrolled in private practice gastroenterology settings from the Franche-Comté region (France) between March 2004 and December 2004. With 4 exceptions, all gastroenterology practices (n = 26) agreed to recruit patients for the study cohort. The initial enrollment criteria included individuals with a first-time diagnosis of histologically confirmed polyps removed during a diagnostic or screening colonoscopy, aged over 18 years. Ineligible patients included those with a prior history of colon disease or an adenoma containing an invasive carcinoma at index colonoscopy.

The baseline bowel preparation was described as good in 95.4% of patients, fair but adequate in 3.3%, and poor in 1.3%. All parts of the colon were thoroughly examined, all polyps were removed regardless of size, by means of a polypectomy snare or hot forceps, and no macroscopically visible polyp tissue was left. The endoscopists numbered the polyps and described their location according to standardized guidelines (cecum and ascending colon, transverse colon, descending colon, sigmoid colon and rectum). They forwarded each removed polyp to the pathological laboratory of their choice (among eight local laboratories).

The endoscopists determined the time of the next examination, depending on the baseline pathology: 10 years in patients with hyperplastic polyps, 5 years in patients with 1 or 2 small (<10 mm) tubular adenomas, or 3 years in patients with advanced neoplasia or more than 2 adenomas.

### Polyp Classification

Pathology reports were reviewed by a trained research assistant to abstract data on the number, size, shape, and location of all colorectal polyps. Histologic features were separated into the following categories: hyperplastic polyp, serrated adenoma, tubular adenoma (≤25% villous component), tubulovillous adenoma (26%–75% villous component), and villous adenoma (>75% villous component). Advanced colorectal adenomas were defined as follows: tubular adenomas ≥10 mm; tubulovillous, villous or serrated adenomas; adenomas with severe dysplasia; or noninvasive carcinomas. Small (<10 mm) tubular adenomas with low-grade dysplasia were classified as non-advanced adenomas.

### Exposure Assessment

At enrollment, interviews were conducted by the physician to obtain data on demographic variables (age and gender) and to measure the purported risk factors for colon neoplasia (including a personal history of non-colorectal cancer, a family history of colorectal polyps or cancer in first-degree relatives), cigarette smoking status, and the use of medication (aspirin, non-selective nonsteroidal anti-inflammatory drugs [NSAIDs] and COX-2-selective NSAIDs). Body mass index (BMI) was calculated from self-reported weight and height. The indications for colonoscopy were recorded. For patients with multiple baseline polyps, the polyp of maximum diameter was used to classify the size and anatomical site.

### Outcome Assessment

Active follow-up was performed through repeat visits to the endoscopists' practices by the research assistant to collect the surveillance colonoscopy reports and abstract them in a standardized fashion. We considered as end points colorectal polyps that were diagnosed at the first surveillance colonoscopic examination. Patients with multiple polyps were classified according to occurrence of the most advanced histologic lesion using the following hierarchy: hyperplastic polyp, non-advanced adenoma, or advanced adenoma.

### Statistical Analysis

All individual data and endoscopic procedures were prospectively recorded on a comprehensive, secure, electronic database. Patients were followed until December 31, 2010. We compared the demographic and lifestyle characteristics of patients followed up (having undergone a surveillance colonoscopy during the study period) and lost to follow up using the t test and chi-square tests for continuous and categorical variables, respectively.

Patients had different durations of follow up (minimum, 0.22 years; maximum, 6.62 years) depending on the gastroenterologist decision and the patient request. Moreover, the point at which a polyp occurred during surveillance could not be observed directly but could only be located between two colonoscopies. This made it impossible to use a simple design to estimate the associations over a fixed time period. To account for these interval-censored observations, the time to event was analyzed with an accelerated failure time model assuming a Weibull distribution. This modeling method expresses the association in terms of hazard ratios (HRs). An HR greater than one indicates that the presence of the covariate decreases the expected duration until polyp recurrence, or in other words, it increases the hazard of polyp recurrence. Conversely, an HR less than one corresponds to a lower hazard (and, thus, to a longer expected duration).

In the Weibull regression models, age, BMI, the number of polyps, and the size of the largest polyp were entered as continuous variables. The baseline polyp location was categorized as distal (including the rectum, sigmoid colon and descending colon) and proximal (including the transverse colon, ascending colon and cecum). Study participants were classified into never, former, and current smokers. All other risk factors for polyp recurrence were binary variables. We first introduced each independent variable in turn in the regression model (univariate approach). In a second step, all variables that had a p value<0.20 were considered simultaneously in a multivariate model. We conducted a secondary analysis on the subset of adenomas diagnosed at baseline with the recurrence of any adenoma during the post-polypectomy surveillance as an end point. The HRs are provided along with the 95% CIs. All p values are 2-sided.

The Weibull regression models were performed using R 2.12.2 statistical software (survival 2.36–5 package) (R Development Core Team, Vienna, Austria, 2011).

### Ethics

Ethical clearance for this study was granted by the Consulting Committee for the Treatment of Information in Medical Research (no. 03.175) and the National Commission for the Confidentiality of Computerized Data (no. 903317). Written informed consent was obtained from each participant.

## Results

### Baseline Characteristics

The demographic and lifestyle characteristics of the study population are given in Table 1. Of the 1023 eligible patients, 553 (54.1%) were females and 470 (45.9) were males. The mean age at the index colonoscopy was 59.7 (standard deviation [SD], 11.9) years.

**Table 1:** Patient demographic and lifestyle characteristics at enrollment (n = 1023).

Characteristic	No. of patients (%)
Gender	
Female	553 (54.1)
Male	470 (45.9)
Age (years)	
<40	49 (4.8)
40–49	172 (16.8)
50–59	311 (30.4)
60–69	267 (26.1)
≥ 70	224 (21.9)
Drug use	
Aspirin	
no	844 (82.5)
yes	80 (7.8)
unknown	99 (9.7)
Non-selective NSAID*	
no	899 (87.9)
yes	23 (2.2)
unknown	101 (9.9)
COX-2-selective NSAID	
no	912 (89.1)
yes	9 (0.9)
unknown	102 (10.0)
Smoking status	
never	553 (54.1)
former	199 (19.4)
current	153 (15.0)
unknown	118 (11.5)
Body mass index (kg/m^2^)	
<25	355 (34.7)
25–29	310 (30.3)
≥ 30	111 (10.9)
unknown	247 (24.1)

* Nonsteroidal anti-inflammatory drug.

The clinical characteristics of patients at the time of enrollment are reported in Table 2. More than one indication for colonoscopy was listed for some patients. The vast majority of colonoscopies were performed for diagnostic purposes (rectal bleeding, 29.2%; abdominal pain, 53.5%; motility disturbances, 48.4%). A family history of polyps (28.0%) or cancer (16.0%) represented the reasons for screening. At baseline, a single polyp was found in 639 (62.5%) patients and multiple polyps were identified in 384 (37.5%) patients.

**Table 2:** Patient clinical characteristics at enrollment (n = 1023).

Characteristic	No. of patients (%)
Indications for colonoscopy*	
Family history of colorectal polyps	
no	788 (77.0)
yes	164 (16.0)
unknown	71 (7.0)
Family history of colorectal cancer	
no	684 (66.9)
yes	287 (28.0)
unknown	52 (5.1)
Rectal bleeding	
no	724 (70.8)
yes	299 (29.2)
Abdominal pain	
no	476 (46.5)
yes	547 (53.5)
Motility disturbance	
no	528 (51.6)
yes	495 (48.4)
No. of polyps	
1	639 (62.5)
2	240 (23.5)
3	90 (8.8)
4+	54 (5.2)
Anatomical site of the largest polyp	
cecum and ascending colon	189 (18.5)
transverse colon	93 (9.1)
descending colon	97 (9.5)
sigmoid colon and rectum	644 (62.9)
Size of the largest polyp (cm)	
<0.5	343 (33.5)
0.5–0.9	425 (41.5)
1.0–1.9	190 (18.6)
≥ 2	65 (6.4)

* Multiple indications could be listed.

The histopathological data of the 1662 polyps removed at the index colonoscopy are listed in Table 3. The most frequent histological types were tubular adenomas and hyperplastic polyps (42.5% and 35.4%, respectively). The polyps were mainly located in the sigmoid colon and rectum (61.4%), although they were also frequent in the less-accessible cecum and ascending colon (17.6%). The vast majority (81.3%) were smaller than 10 mm in diameter. Of the 1074 adenomas, 1016 (94.6%) were classified as low grade (mild or moderate dysplasia).

**Table 3:** Characteristics of colorectal polyps at index colonoscopy (n = 1662).

Characteristic	No. of polyps (%)
Histology	
hyperplastic polyp	588 (35.4)
serrated adenoma	21 (1.3)
tubular adenoma	707 (42.5)
tubulovillous adenoma	289 (17.4)
villous adenoma	41 (2.4)
unspecified*	16 (1.0)
Classification	
hyperplastic polyp	588 (35.4)
non-advanced adenoma	578 (34.8)
advanced adenoma	496 (29.8)
Anatomical site	
cecum and ascending colon	292 (17.6)
transverse colon	165 (9.9)
descending colon	184 (11.1)
sigmoid colon and rectum	1021 (61.4)
Shape	
pedunculated	313 (18.8)
sessile	1309 (78.8)
flat	40 (2.4)
Size (cm)	
<0.5	694 (41.8)
0.5–0.9	657 (39.5)
1.0–1.9	243 (14.6)
≥ 2	68 (4.1)
Degree of dysplasia	
mild	615 (37.0)
moderate	401 (24.1)
severe	40 (2.4)
non-invasive carcinoma	18 (1.1)
not applicable†	588 (35.4)

* Other polyps include lymphoid aggregates and hamartoma.

† Hyperplastic polyps.

**Figure 1:**
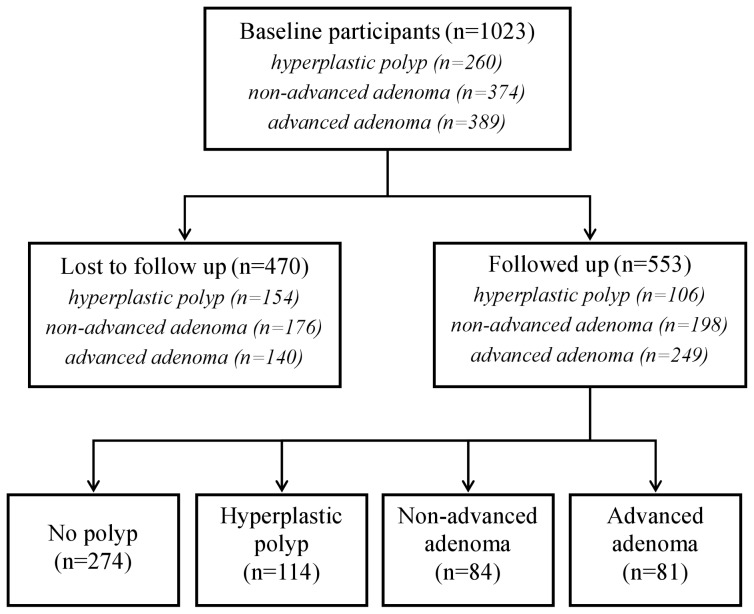
Flow diagram for the colorectal polyp recurrence study.

Among the 1023 patients with colorectal polyps at baseline, 553 underwent a follow up colonoscopy (Figure 1). They were similar to those who did not have colonoscopic surveillance with regard to gender (p = 0.89), drug use (aspirin: p = 0.48, non-selective NSAID: p = 0.79; COX2-selective-NSAID: p = 0.19), smoking status (p = 0.10), BMI (p = 0.45), a family history of colorectal polyp (p = 0.07), rectal bleeding (p = 0.23), or motility disturbance (p = 0.09). However, patients who completed the follow up were younger (p = 0.01), suffered more frequent abdominal pain (p<0.01), and more frequently had a family history of colorectal cancer (p<0.0001).

### Follow Up

The mean time interval from baseline colonoscopy to first surveillance examination was 3.42 (SD, 1.45) years. When considering each histological type separately, the mean follow up periods were 4.22 (SD, 1.43), 3.58 (SD, 1.19), and 2.95 (SD, 1.47) years for hyperplastic polyp (n = 106), non-advanced adenoma (n = 198), and advanced adenoma (n = 249), respectively. No CRC cases were identified in the surveillance population.

### Recurrent Polyps

During the follow up evaluation of the 553 patients, hyperplastic polyps were found in 114 patients (20.6%), non-advanced adenomas were found in 84 patients (15.2%), and advanced adenomas were found in 81 patients (14.7%) (Figure 1). Colorectal polyps were not identified in the remaining 274 patients (49.5%).

Among all baseline patient and polyp characteristics considered, only 5 had a p value<0.20 in a univariate approach, and only 3 of them were statistically significant (Table 4). Male patients had a significantly lower risk of developing new polyps than female patients (HR 0.61, 95% CI 0.47 to 0.80). Body mass index and the number of polyps were associated with a faster rate of polyp recurrence (HR 1.22 per 5 kg/m^2^, 95% CI 1.04 to 1.42; HR 1.18 per 1 increase, 95% CI 1.07 to 1.30, respectively). In the multivariate model, the number of polyps at baseline remained the only significant predictor for recurrence (HR 1.19 per 1 increase, 95% CI 1.06 to 1.33) (Table 4). A positive trend, although not statistically significant (p = 0.07), was noticeable for smoking status.

**Table 4:** Hazard ratios for polyp recurrence, according to baseline patient and polyp characteristics (553 followed up participants).

	Univariate analysis*			Multivariate analysis		
Characteristic	Hazard ratio	95% CI	*P* value	Hazard ratio	95% CI	*P* value
Gender						
female	1	reference		1	reference	
male	0.61	0.47 to 0.80	<10^−3^	0.76	0.56 to 1.04	0.09
Aspirin use						
no	1	reference		1	reference	
yes	1.44	0.92 to 2.24	0.11	1.10	0.65 to 1.87	0.72
Smoking status						
never	1	reference		1	reference	
former	1.33	0.97 to 1.83	0.07	1.10	0.77 to 1.56	0.60
current	1.35	0.95 to 1.91	0.10	1.45	0.99 to 2.14	0.06
Body mass index (per 5 kg/m^2^)	1.22	1.04 to 1.42	0.02	1.16	0.98 to 1.37	0.09
Number (per 1 increase)	1.18	1.07 to 1.30	<10^−3^	1.19	1.06 to 1.33	<10^−2^

* variables that had a p value<0.20.

### Recurrent Adenomas

**Figure 2:**
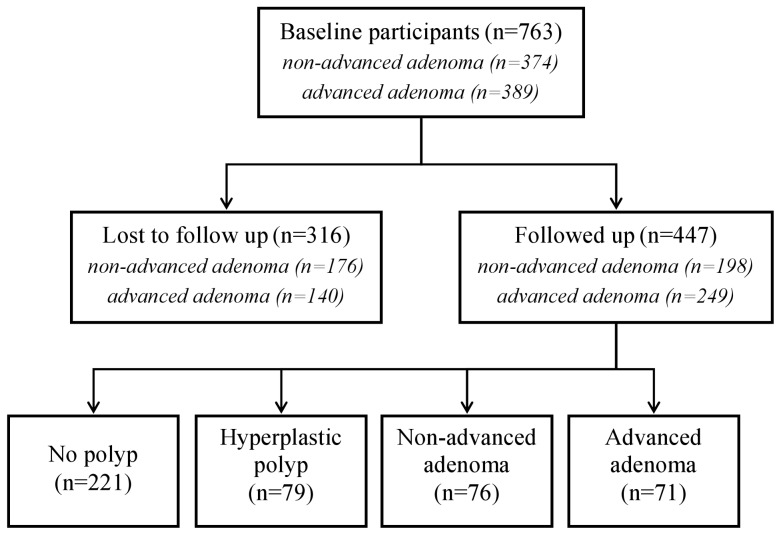
Flow diagram for the colorectal adenoma recurrence study.

Considering only the 447 patients with a colorectal adenoma at the initial clearing procedure, the adenoma recurrence rate was 32.9% (non-advanced adenomas, 17.0%; advanced adenomas, 15.9%) (Figure 2). The remaining participants either had a hyperplastic polyp (17.7%) or neither outcome (49.4%).

Five baseline patient and adenoma characteristics had a p value<0.20 in a univariate analysis, and 2 were statistically significant (Table 5): male gender delaying adenoma recurrence (HR 0.63, 95% CI 0.44 to 0.90) and the number of adenomas shortening the recurrence time (HR 1.20 per 1 increase, 95% CI 1.06 to 1.35). The latter remained the only independent predictor of a recurrent adenoma in the multivariate approach (HR 1.17 per 1 increase, 95% CI 1.03 to 1.34) (Table 5).

**Table 5:** Hazard ratios for adenoma recurrence, according to baseline patient and adenoma characteristics (447 followed up participants).

	Univariate analysis*			Multivariate analysis		
Characteristic	Hazard ratio	95% CI	*P* value	Hazard ratio	95% CI	*P* value
Gender						
female	1	reference		1	reference	
male	0.63	0.44 to 0.90	0.01	0.71	0.48 to 1.04	0.08
Age (per 10 years)	1.12	0.95 to 1.31	0.16	1.07	0.89 to 1.28	0.48
Aspirin use						
no	1	reference		1	reference	
yes	1.68	0.97 to 2.92	0.07	1.38	0.77 to 2.48	0.27
Number (per 1 increase)	1.20	1.06 to 1.35	<10^−2^	1.17	1.03 to 1.34	0.01
Advanced adenoma						
no	1	reference		1	reference	
yes	1.32	0.95 to 1.84	0.10	1.32	0.93 to 1.88	0.12

* variables that had a p value<0.20.

## Discussion

This private practice-based study was conducted to gain a better understanding of how surveillance colonoscopy is being used in the community, the types of lesions found in subsequent examinations, and the subsequent patient outcomes. This study revealed that, in a European community population, the only baseline polyp characteristic associated with a mean 3.42 year recurrence was the number of polyps. Conversely, other polyp features (anatomical site and size) and patient characteristics did not predict recurrence in a multivariate approach.

The strengths of this study arise from its prospective design and its community-based feature. Community endoscopists of varied experience and expertise performed all of the study colonoscopies for a variety of indications and were the ultimate determinant of surveillance use. Because in France specialized clinicians are mainly consulted by referral from general practitioners (through a process known as coordinated care), the possibility that some patients were followed up by an endoscopist not involved in the study appears unlikely. Most participants were symptomatic and not particularly health conscious. This “real-world” setting ensures the generalizability and applicability of our findings. One other strength consists of the sound and flexible statistical technique we used, accounting for the interval-censored observations and allowing for the associated hazard rate to vary with respect to time. To the best of our knowledge, only one publication has so far considered interval censoring with respect to the event of interest [1]. Finally, a comprehensive range of patient and polyp characteristics was considered, resulting in a more detailed risk analysis through multivariate adjustments.

Some limitations must be considered in interpreting our results. The mean 3.42 year duration of follow up evaluation is relatively short, as this study focuses on the findings of the first surveillance colonoscopy and does not consider subsequent recurrences in a long-term follow up study. Although of sufficient statistical power to highlight an increased risk with polyp multiplicity, our study population size did not allow us to generate more precise hazard ratio estimates for other risk factors in the multivariate analyses. The histopathologic examination was performed by 8 different pathology laboratories, which may increase the potential for polyp misclassification. If relevant, it would be expected to be nondifferential with respect to polyp recurrence, such that the true associations may even have been underestimated. Colonoscopy quality may be an important factor in routine clinical practice, and it is not known how many lesions are true new lesions and how many represent polyps missed during the initial colonoscopy. The miss rate of polyps at colonoscopy has been evaluated in several tandem studies and is approximately 20% [8]. However, the majority of these lesions are small and thought to be of low clinical importance.

Although the current recommendations suggest that patients with hyperplastic polyps at baseline undergo a surveillance examination every 10 years (because hyperplastic polyps traditionally have been considered to have little malignant potential), 106 of the 260 patients in this study underwent a surveillance colonoscopy within 4.22 years on average. Conversely, as reported in another community practice study [9], a significant proportion of subjects with non-advanced adenomas (176 of 374) or advanced adenomas (140 of 389) did not undergo a surveillance colonoscopy. Both results are in line with the growing body of evidence indicating the overuse of surveillance colonoscopy among low-risk subjects and the underuse among high-risk subjects [10]. Many factors may influence surveillance use, including physician preference and patient behavior. Physicians tend to recommend the surveillance of hyperplastic polyps in excess of the published guidelines [10]–[13]. In agreement with Brueckl et al. who showed that no symptoms and old age were reasons for non-compliance [14], we identified the absence of abdominal pain, the absence of a family history of colorectal cancer, and old age as predictors of non-compliance in surveillance.

No CRC occurred during follow up, most likely in relation with the limited size of the surveillance population (553 participants), and the local incidence rates of CRC observed in 2004 (33.4/100000 in males, 18.9/100000 in females). The adenoma recurrence rate found in this study (32.9%) is of similar magnitude to those reported in previous studies [2], [15]–[20], suggesting that colorectal polyps are not missed more frequently in the community setting than in the academic setting. However, this recurrence rate is somewhat higher than the rate reported in an intervention trial conducted in a contiguous French region (22.1%) [21].

Our findings corroborate those of several other studies demonstrating the number of removed polyps at baseline examination to be a consistent predictor of polyp recurrence. Multiplicity as a risk factor has been implicated in numerous other studies (see [5] for a review and [6] for a meta-analysis) and most likely defines individuals with a propensity to a faster rate of polyp growth. Associations between recurrence rates and the anatomical site or maximum polyp size were not found (unlike many other reports), nor were relationships demonstrated with gastrointestinal complaints.

The identification of risk factors that influence the rate of colorectal polyp development is important because they represent modifiable factors for targeted risk reduction in at-risk individuals. We found that the risk of polyp recurrence is lower among men than women in a univariate approach, which is at odds with previous results [1], [22], [23], although possible explanations include our population-based sample (where males are not overrepresented as in many surveillance studies) and the adjustment for smoking status. We found a linear trend of borderline significance between smoking status and polyp recurrence, consistent with previous reports focusing on the subsequent development of adenomas [24], or hyperplastic polyps [25]. Contradictory evidence has been reported on aspirin use. A recent meta-analysis of randomized controlled trials suggested that low-dose aspirin modestly reduces adenoma recurrence [26]. Unlike this finding, we showed that self-reported aspirin use (of unknown frequency or quantity) was modestly associated with polyp recurrence but only in a univariate approach. This apparent discordance in results may reflect differences in the extent to which risk factors were taken into account in the various reports. Our study adds to the previous data [27], supporting obesity as a risk factor for the subsequent short-interval development of colorectal polyps in a univariate analysis.

In summary, this population-based study evaluated the use and yield of surveillance colonoscopy under real circumstances in clinical practice, filling the knowledge gap between academic and community practice. The efficacy of surveillance colonoscopy in community gastroenterology practice compared favorably with academic settings, as assessed by the similar magnitude of recurrence rates. This study provides further evidence that the number of initial colorectal polyps is useful for predicting the risk of polyp recurrence, even in the community setting.
